# Supplementary material for: p21 regulates expression of ECM components and promotes pulmonary fibrosis via CDK4 and Rb
Source: EMBO J. 2024 Sep 30;43(22):5360–80. doi: 10.1038/s44318-024-00246-7 (PMC11574164; doi:10.1038/s44318-024-00246-7)
Supplement: Supplementary file 1 — Appendix [file 44318_2024_246_MOESM1_ESM.pdf]

Appendix

p21 regulates expression of ECM components and promotes pulmonary fibrosis via CDK4 and Rb

Nurit Papismadov[1], Naama Levi[1], Lior Roitman[1], Amit Agrawal[1], Yossi Ovadya[1], Ulysse Cherqui[1], Reut Yosef[1], Hagay Akiva[1], Hilah Gal[1] and Valery Krizhanovsky[1,*]

[1]Department of Molecular Cell Biology, The Weizmann Institute of Science, 7610001 Rehovot, Israel

*****Corresponding author: valery.krizhanovsky@weizmann.ac.il

Valery Krizhanovsky

Department of Molecular Cell Biology

The Weizmann Institute of Science

7610001 Rehovot, Israel

Tel: +972-8-934-6575; Fax: +972-8-934-4125

Table of contents

Appendix Figure S1 3

Appendix Figure S2 5

Appendix Figure S3 7

Appendix Figure S4 8

Appendix Figure S5 10

Appendix Figure S6 11

Appendix Figure S7 13

Appendix Figure S8 14

Appendix Figure S9 16

Appendix Figure S10 17

Appendix Figure S11 18

References 19

Appendix Figure S1

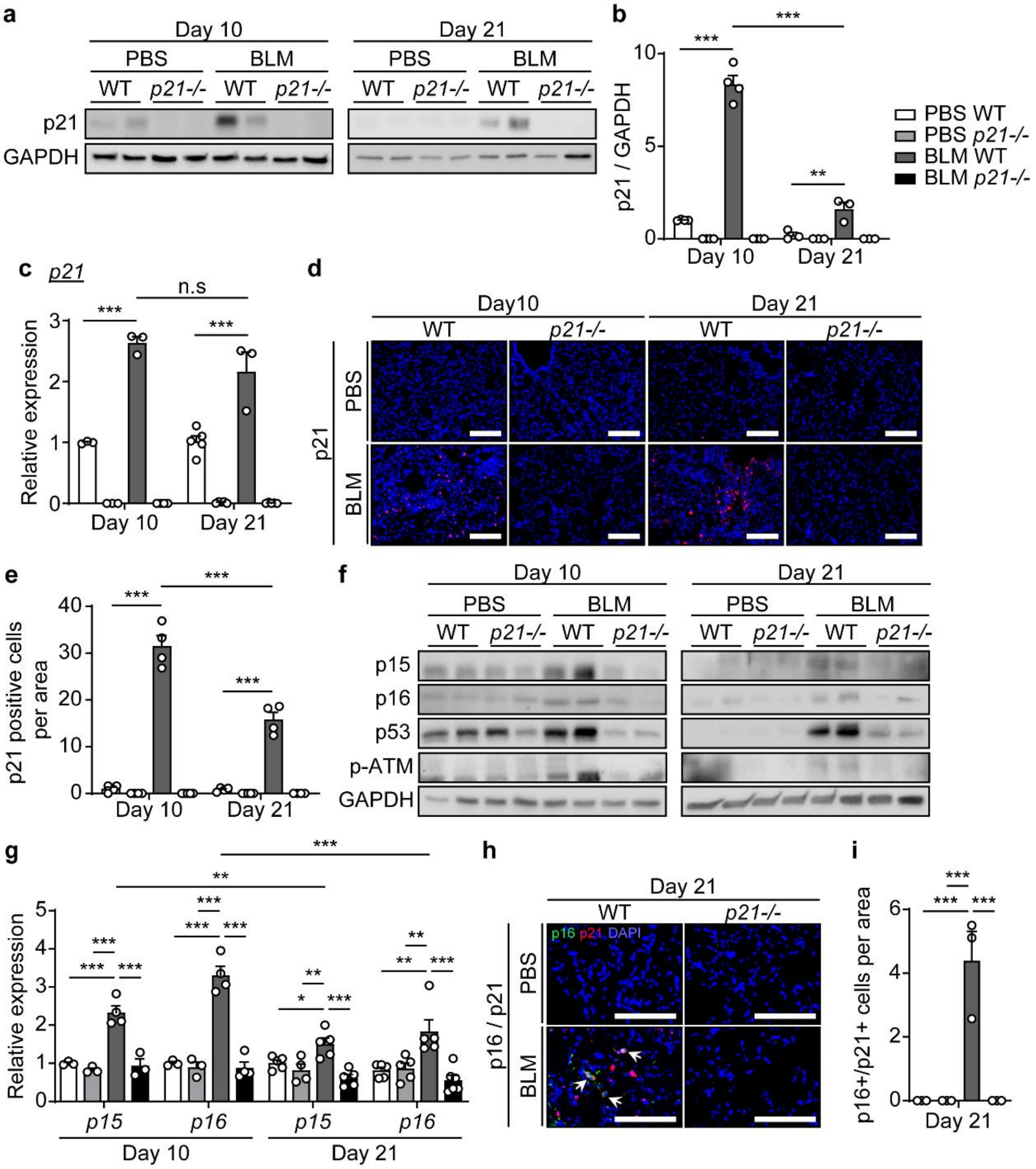

Appendix Figure S1: p21 knockout leads to a reduction in the presence of senescent markers in bleomycin-induced lung fibrosis. Related to figure 1. WT or *p21*$^{-/-}$ mice were administered with bleomycin (BLM) or PBS vehicle by one intra-tracheal installation. The lungs were analyzed 10 and 21 days post BLM administration. (a) Western blot analysis of p21 in the mice lungs. (b) Quantification of protein levels of p21 relative to PBS controls presented in (a) (BLM WT Day 10 vs. BLM WT Day 21, p21 [p<0.0001]). (c) mRNA expression levels of *p21* relative to control in the mice lungs (BLM WT Day 10 vs. BLM WT Day 21, *p21* [p<0.0001]). (d) IF staining of p21 relative to PBS controls. Scale bar: 200µm. (e) Quantification of p21 IF staining presented in (d) (BLM WT Day 10 vs. BLM WT Day 21, p21 [p<0.0001]). (f) Western blot analysis of p15,

p16, and p53, and phospho-ATM (p-ATM) in mice lungs. (g) mRNA expression levels of *p15* and *p16* relative to PBS controls (BLM WT Day 10 vs. BLM WT Day 21, *p15* [p<0.002], *p16* [p<0.0001]). (h) IF co-staining of p16 (green) and p21 (red) positive cells relative to PBS controls. Scale bar: 200µm. (i) Quantification of p16 p21 positive cells in the IF co-staining presented in (h) (WT BLM vs. BLM $p21^{-/-}$, p=0.0001). Data information: Data were analyzed using one-way ANOVA. *P<0.05. **P<0.005. ***P<0.0005. Data are presented as mean ±SEM (a-b, n=3-4; c, n=3-5; d-e, n=4-5; f, n=4-6; g, n=3-6; h-i, n=3 independent repeats).

Appendix Figure S2

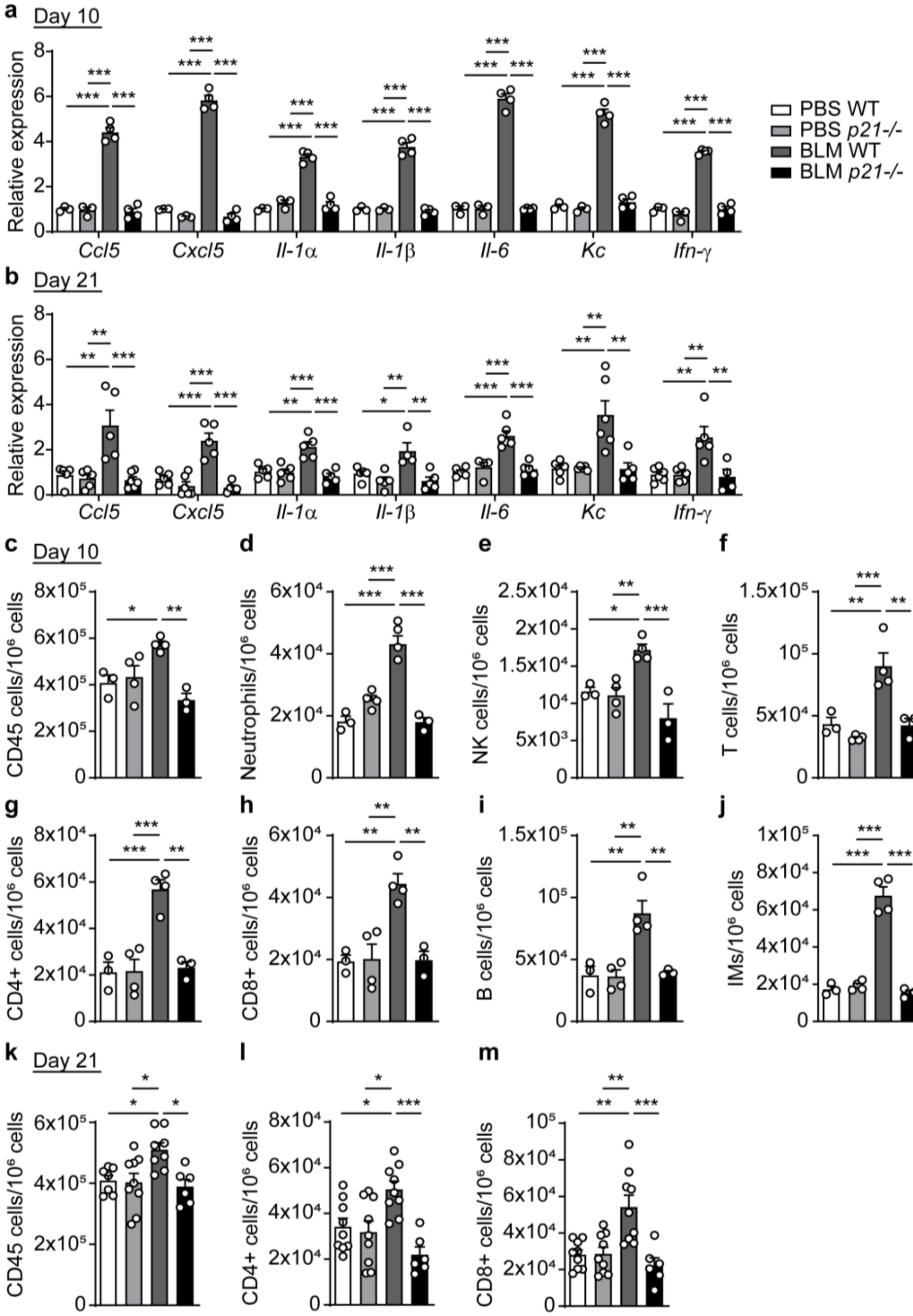

Appendix Figure S2: p21 knockout reduces the inflammatory responses in bleomycin-induced lung fibrosis. Related to figure 1. WT or *p21*$^{-/-}$ mice were administered with bleomycin (BLM) or PBS vehicle. The lungs were analyzed 10 and 21 days thereafter. (a-b) mRNA expression levels of the indicated cytokines relative to control in the mice lungs (a) 10 days and (b) 21 days following BLM administration. (c-j) Single-cell suspensions of WT and *p21*$^{-/-}$ mice lungs

10 days post BLM administration were analyzed by flow-cytometry for numbers of (c) immune cells (CD45+), (d) neutrophils (CD45+/Ly6G+/CD11b+), (e) NK cells (CD45+/Ly6G-/Nkp46+), (f) T cells (CD45+/CD3+), (g) CD4+ T cells (CD45+/CD3+/CD4+), (h) CD8+ T cells (CD45+/CD3+/CD8+), (i) B cells (CD45+/B220+) and (j) interstitial macrophages (IM's) (CD45+/CD11c+/SiglecF-/CD11b+/CD24+) (WT BLM vs. BLM *p21*$^{-/-}$, CD45+ [p=0.003], neutrophils [p<0.0001], NK cells [p=0.0009], T cells [p=0.004], CD4+ T cells [p=0.001], CD8+ T cells [p=0.004], B cells [p=0.005], IM's [p<0.0001]). (k-m) Single-cell suspensions of WT and *p21*$^{-/-}$ mice lungs 21 days post BLM administration were analyzed by flow-cytometry for numbers of (k) immune cells (CD45+), (l) CD4+ T cells (CD45+/CD3+/CD4+), and (m) CD8+ T cells (CD45+/CD3+/CD8+) +) (WT BLM vs. BLM *p21*$^{-/-}$, CD45+ [p=0.038], CD4+ T cells [p=0.0004], CD8+ T cells [p=0.0004]). Data information: Data were analyzed using one-way ANOVA. *P<0.05. **P<0.005. ***P<0.0005. Data are presented as mean ±SEM (a, n=3-4; b, n=4-6; c-j, n=3-4; k-m, n=6-9 independent repeats).

Appendix Figure S3

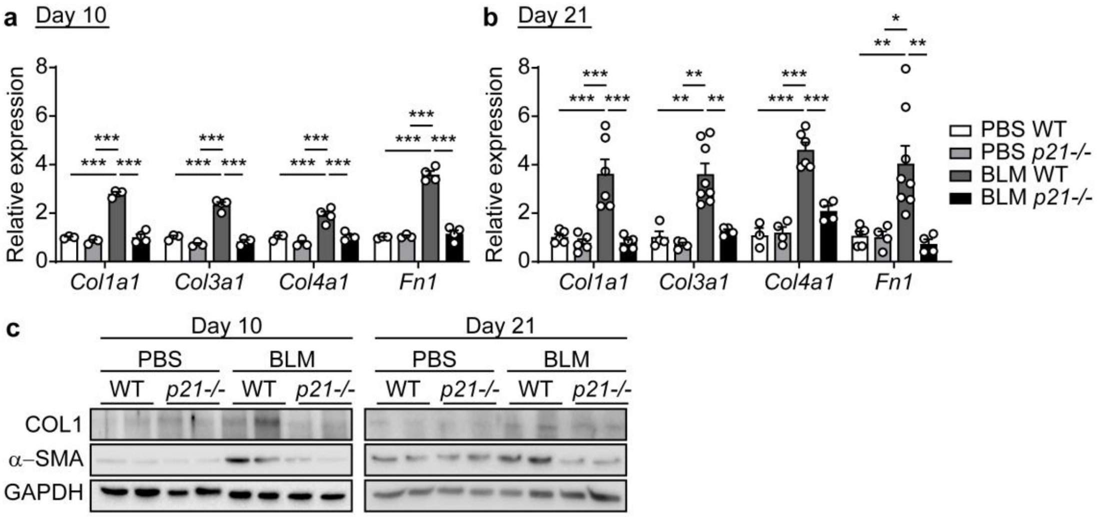

Appendix Figure S3: p21 knockout reduces ECM components expression in bleomycin-induced lung fibrosis. Related to figure 2. WT or *p21*$^{-/-}$ mice were administered with bleomycin (BLM) or PBS vehicle. The lungs were analyzed 10 and 21 days thereafter. (a-b) mRNA expression levels collagen-1 (*Col1a1*), collagen-3 (*Col3a1*), collagen-4 (*Col4a1*) and Fibronectin-1 (*Fn1*) relative to PBS controls in the mice lungs (a) 10 days and (b) 21 days following BLM administration (WT BLM vs. BLM *p21*$^{-/-}$ day 10, *Col1a1* [p<0.0001], *Col3a1* [p<0.0001], *Col4a1* [p=0.0007], *Fn1* [p<0.0001]); day 21, *Col1a1* [p=0.0001], *Col3a1* [p=0.007], *Col4a1* [p<0.0001], *Fn1* [p=0.005]). (c) Western blot analysis of collagen-1 (COL1) and α-SMA in mice lungs. Data information: Data were analyzed using one-way ANOVA. *P<0.05. **P<0.005. ***P<0.0005. Data are presented as mean ±SEM (a, n=3; b, n=3-8; c, n=4-9 independent repeats).

Appendix Figure S4

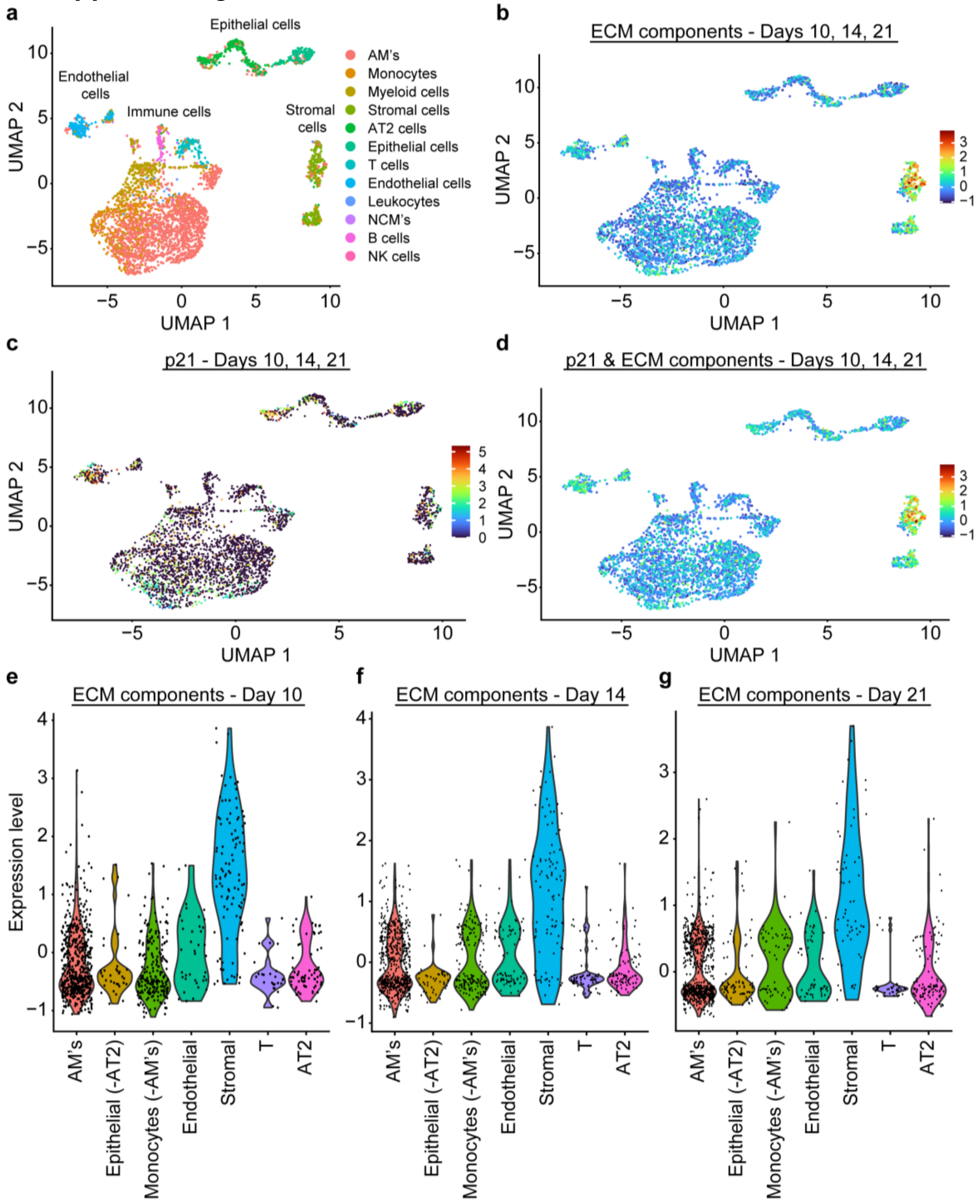

Appendix Figure S4: scRNAseq analysis of p21 and ECM components expression at day 10, 14, and 21, in lung cells following BLM administration. Related to figure 2. Analysis of scRNA-seq data from Strunz et al. [1] (GEO accession code GSE141259) of female mice (WT) lung cells 10 days (n=3), 14 days (n=4) and 21 days (n=4) following bleomycin (BLM) administration or PBS vehicle (n=7). (a) Uniform manifold approximation and projection (UMAP) plot of the re-clustered lung cells colored based of cell type. (b) UMAP plots of the module score of ECM components

genes (collagen-1 [*Col1a1*], collagen-3 [*Col3a1*], collagen-4 [*Col4a1*] and Fibronectin-1 [*Fn1*]). (c) UMAP plots of the module score of the p21 gene. (d) UMAP plots of the module score of the p21 gene and the indicated ECM components genes. (e-g) Expression of the indicated ECM components genes in alveolar macrophages (AMs), monocytes (-AMs), T cells, Alveolar Type II (AT2) cells, epithelial cells (-AT2 cells), endothelial cells and stromal cells. The ECM components expression was analyzed at (e) day 10, (f) day 14, and (g) day 21 post BLM administration.

Appendix Figure S5

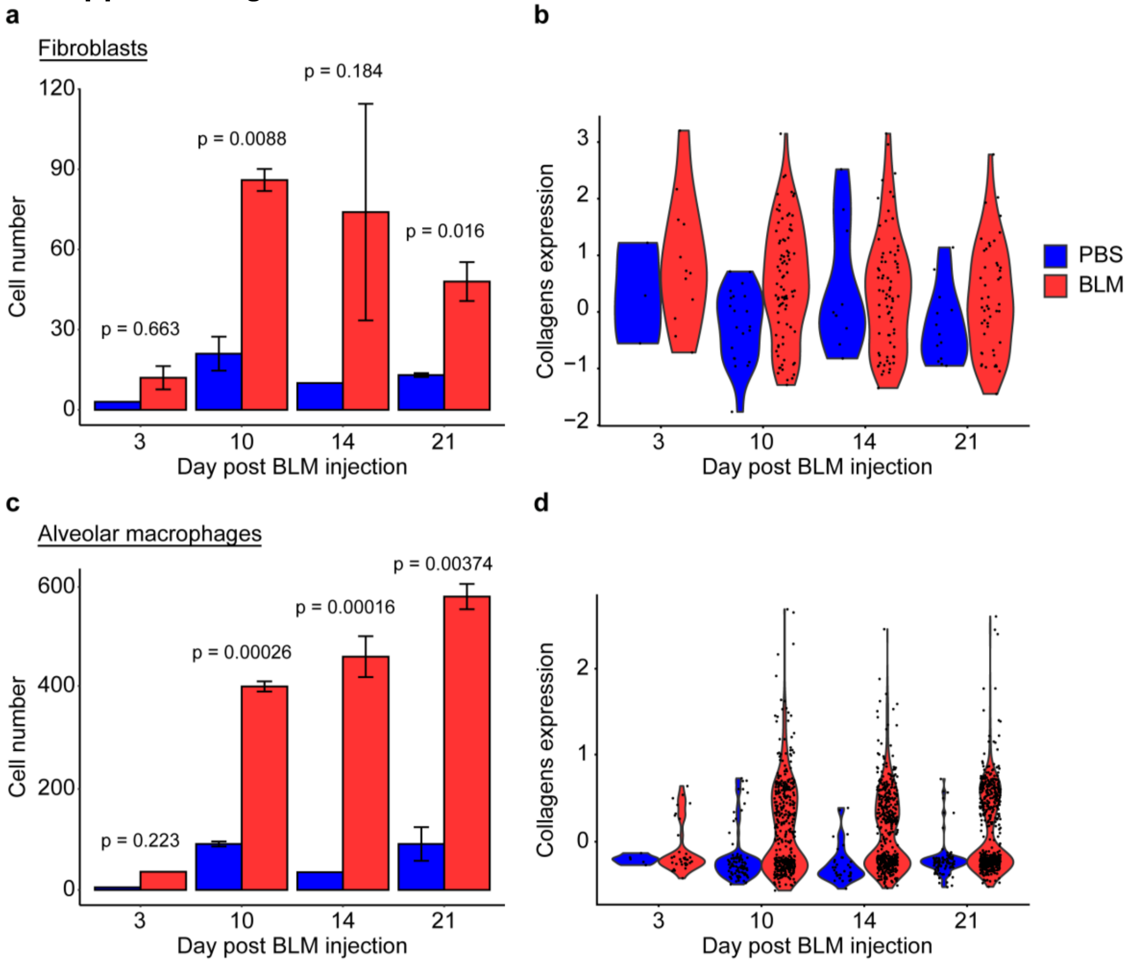

Appendix Figure S5: scRNAseq analysis of fibroblasts and alveolar macrophages in BLM-induced lung fibrosis. Related to figure 2. Analysis of scRNA-seq data from Strunz et al. [1] (GEO accession code GSE141259) of female mice (WT) lung cells 3 days (n=3), 10 days (n=3), 14 days (n=4) and 21 days (n=4) following bleomycin (BLM) administration or PBS vehicle (n=7). (a) Number of fibroblasts 3, 10, 14 and 21 days following BLM administration relative to PBS controls. (b) Module score analysis of the expression of the ECM genes (collagen-1 [*Col1a1*], collagen-3 [*Col3a1*], collagen-4 [*Col4a1*] and Fibronectin-1 [*Fn1*]) in the fibroblasts described in (a). (c) Number of alveolar macrophages 3, 10, 14 and 21 days following BLM administration relative to PBS controls. (d) Module score analysis of the expression of the ECM components genes (collagen-1 [*Col1a1*], collagen-3 [*Col3a1*], collagen-4 [*Col4a1*] and Fibronectin-1 [*Fn1*]) in the alveolar macrophages described in (c). Data information: Data are presented as mean ±SD (a-d, n=3-7 independent repeats).

Appendix Figure S6

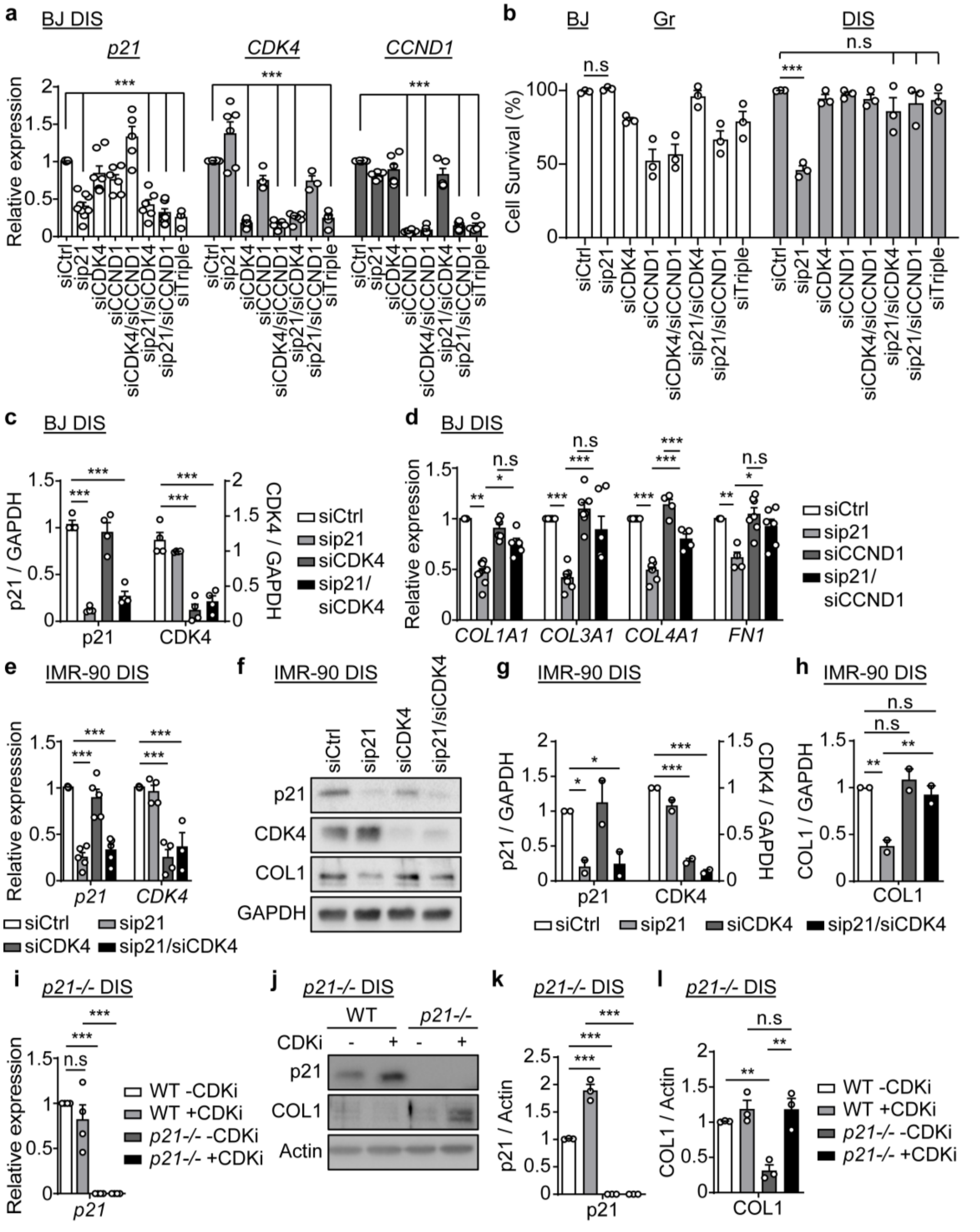

Appendix Figure S6: The differences in the expression of ECM components in senescent cells following p21 knockdown are CDK4-dependent. Related to Figure 3. (a) mRNA expression levels of *p21*, *CDK4* and CyclinD1 (*CCND1*) relative to control in DIS BJ cells following treatment with siRNAs targeting p21 (sip21), CDK4 (siCDK4), CyclinD1 (siCCND1), or their combinations relative to control (siCtrl). (b) Survival of proliferating (Gr) and DIS BJ cells described

in (a) (DIS siCtrl vs. sip21, p=0.0001). (c) Quantification of protein levels of p21 and CDK4 relative to GAPDH control in DIS BJ cells were that transduced with siRNAs targeting p21 (sip21), CDK4 (siCDK4), or control siRNAs (siCtrl) presented in Fig. 3c. (d) mRNA expression levels of collagen-1 (*COL1A1*), collagen-3 (*COL3A1*), collagen-4 (*COL4A1*) and Fibronectin-1 (*FN1*) relative to control in DIS BJ cells following treatment with siRNA targeting p21, CyclinD1, or their combination (sip21 vs. sip21/siCCND1, *COL1A1* [p=0.031], *COL3A1* [p=0.0003], *COL4A1* [p<0.0001], *FN1* [p=0.014]). (e) mRNA expression levels of *p21* and *CDK4* relative to control in DIS IMR-90 fibroblasts following treatment with siRNAs targeting p21, CDK4, or their combination. (f) Western blot analysis of p21, CDK4 and collagen-1 (COL1) in DIS IMR-90 cells described in (e). (g) Quantification of protein levels of p21 and CDK4 relative to GAPDH control DIS IMR-90 cells described in (e). (h) Quantification of protein levels of COL1 relative to GAPDH control DIS IMR-90 cells described in (e) (sip21 vs. sip21/siCDK4, p=0.029). (i) mRNA expression levels of *p21* relative to control in mouse lung fibroblast cells (MLFs) derived from WT or $p21^{-/-}$ mice incubated with or without the CDK4 inhibitor Abemaciclib (+/-CDKi). (j) Western blot analysis of p21 and COL1 in DIS WT and $p21^{-/-}$ MLF cells described in (i). (k) Quantification of protein levels of p21 relative to GAPDH control in DIS WT and $p21^{-/-}$ MLF cells described in (i). (l) Quantification of protein levels of COL1 relative to GAPDH control in DIS WT and $p21^{-/-}$ MLF cells described in (i) ($p21^{-/-}$ -CDKi vs. $p21^{-/-}$ +CDKi , p=0.002). Data information: Data were analyzed using one-way ANOVA. *P<0.05. **P<0.005. ***P<0.0005. Data are presented as mean ±SEM (a, n=3-9; b, n=3; c, n=3; d, n=4-8; e, n=3-6; f-h, n=2; i, n=4; j-l, n=3 independent repeats).

Appendix Figure S7

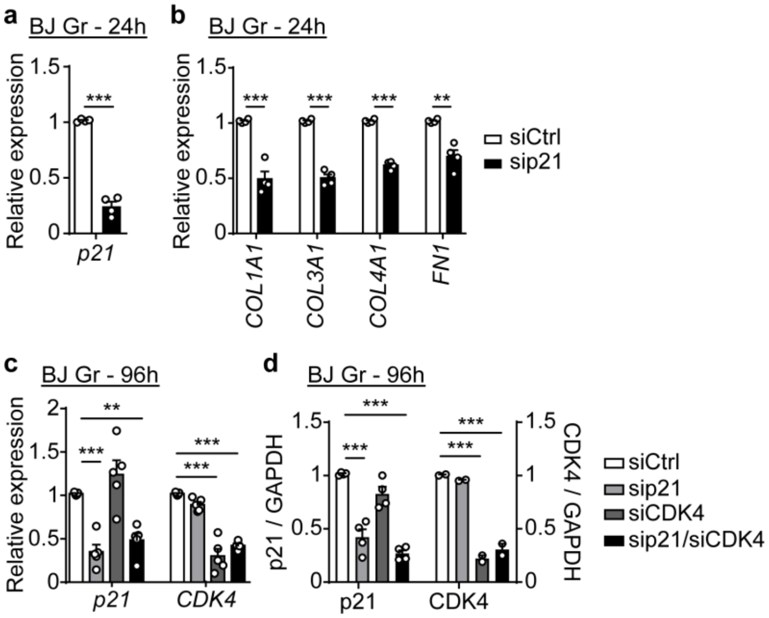

Appendix Figure S7: p21 has a direct effect on the extracellular microenvironment in proliferating cells. Related to Figure 3. (a) mRNA expression levels of *p21* in proliferating (Gr) BJ cells following treatment with siRNA targeting p21 (sip21) for 24h relative to control (siCtrl). (b) mRNA expression levels of collagen-1 (*COL1A1*), collagen-3 (*COL3A1*), collagen-4 (*COL4A1*) and Fibronectin-1 (*FN1*) relative to control in proliferating (Gr) BJ cells described in (a) (siCtrl vs. sip21, *COL1A1* [p=0.0003], *COL3A1* [p<0.0001], *COL4A1* [p<0.0001], *FN1* [p=0.002]). (c) mRNA expression levels of *p21* and *CDK4* relative to control in proliferating BJ cells following treatment with siRNAs targeting p21, CDK4 (siCDK4), or their combination for 96h. (d) Quantification of protein levels of p21 and CDK4 relative to GAPDH control in proliferating (Gr) BJ cells presented in Fig. 3h. Data information: Data were analyzed using one-way ANOVA. *P<0.05. **P<0.005. ***P<0.0005. Data are presented as mean ±SEM (a, n=4; b, n=3-4; c, n=4-5; d, n=4 independent repeats).

Appendix Figure S8

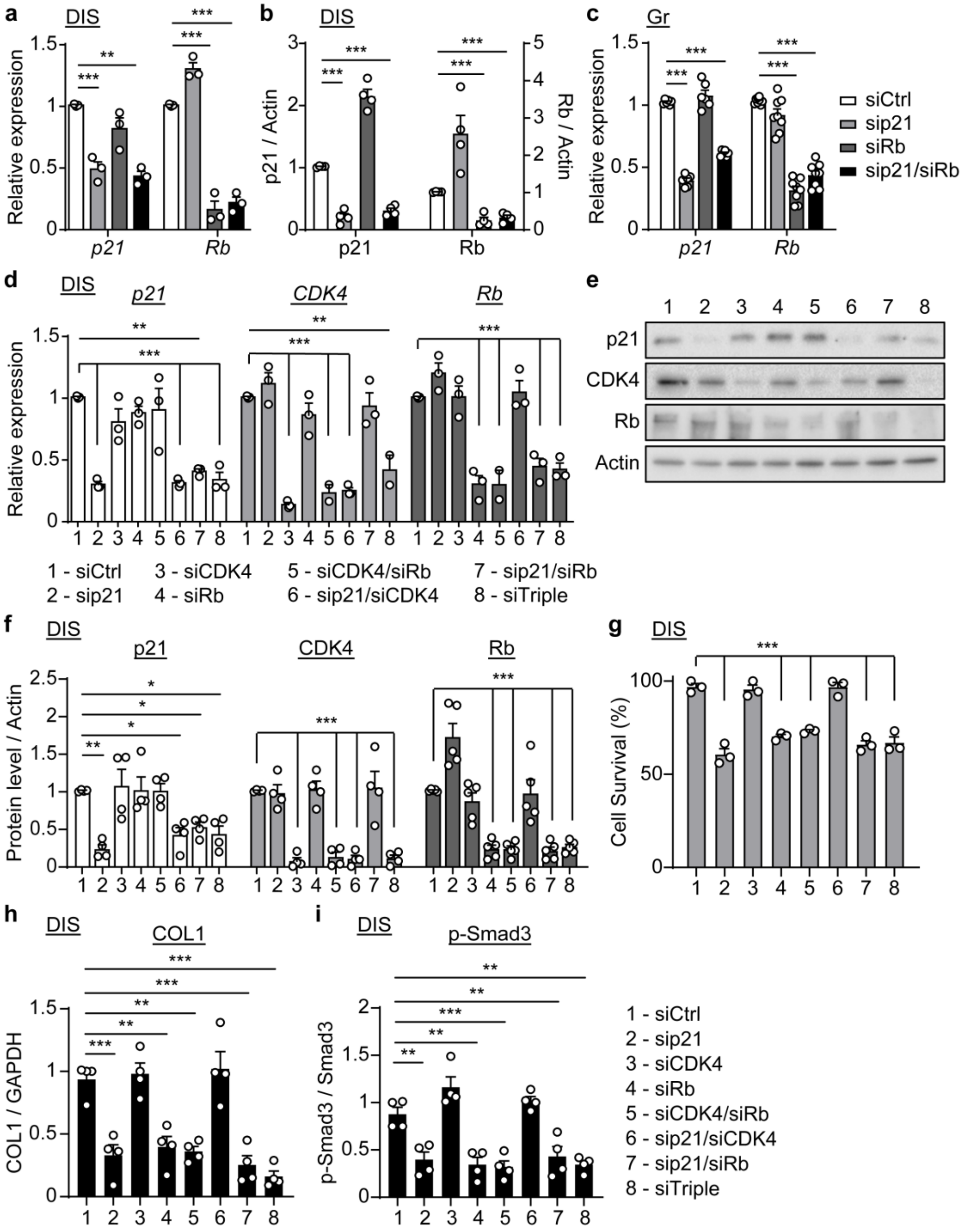

Appendix Figure S8: Rb phosphorylation downstream to p21 regulates ECM components expression via Smad-3. Related to Figure 4. (a) mRNA expression levels of *p21* and *Rb* in DIS BJ cells following treatment with siRNA targeting p21 (sip21), Rb (siRb), or their combination, relative to control (siCtrl). (b) Quantification of protein levels of p21 and Rb relative to GAPDH control in proliferating DIS BJ cells presented in Fig. 4c. (c) mRNA expression levels of *p21* and

Rb in proliferating (Gr) BJ cells following treatment with siRNA targeting p21, Rb, or their combination, relative to control. (d) mRNA expression levels of *p21*, *CDK4* and *Rb* in DIS BJ cells following treatment with siRNA targeting p21, CDK4 (siCDK4), Rb, or their combinations, relative to control (siCtrl). (e) Western blot analysis of p21, CDK4 and Rb in DIS BJ cells described in (d). (f) Quantification of protein levels of p21, CDK4 and Rb relative to Actin control in DIS DIS BJ cells described in (e). (g) Survival of DIS BJ cells described in (d) (siCtrl vs. sip21, p=0.0001). (h) Quantification of protein levels (presented in Fig. 4g) of collagen-1 (COL1) relative to GAPDH control in DIS BJ cells (siCtrl vs. siTriple, p<0.0001). (i) Quantification of protein levels (presented in Fig. 4h) of phospho-Smad3 (p-Smad3) relative to Smad3 DIS BJ cells cells (siCtrl vs. siTriple, p=0.001). Data information: Data were analyzed using one-way ANOVA. *P<0.05. **P<0.005. ***P<0.0005. Data are presented as mean ±SEM (a, n=3; b, n=4; c, n=7-8; d, n=2-3; e-f, n=4-5; g, n=3; h, n=4; i, n=4 independent repeats).

Appendix Figure S9

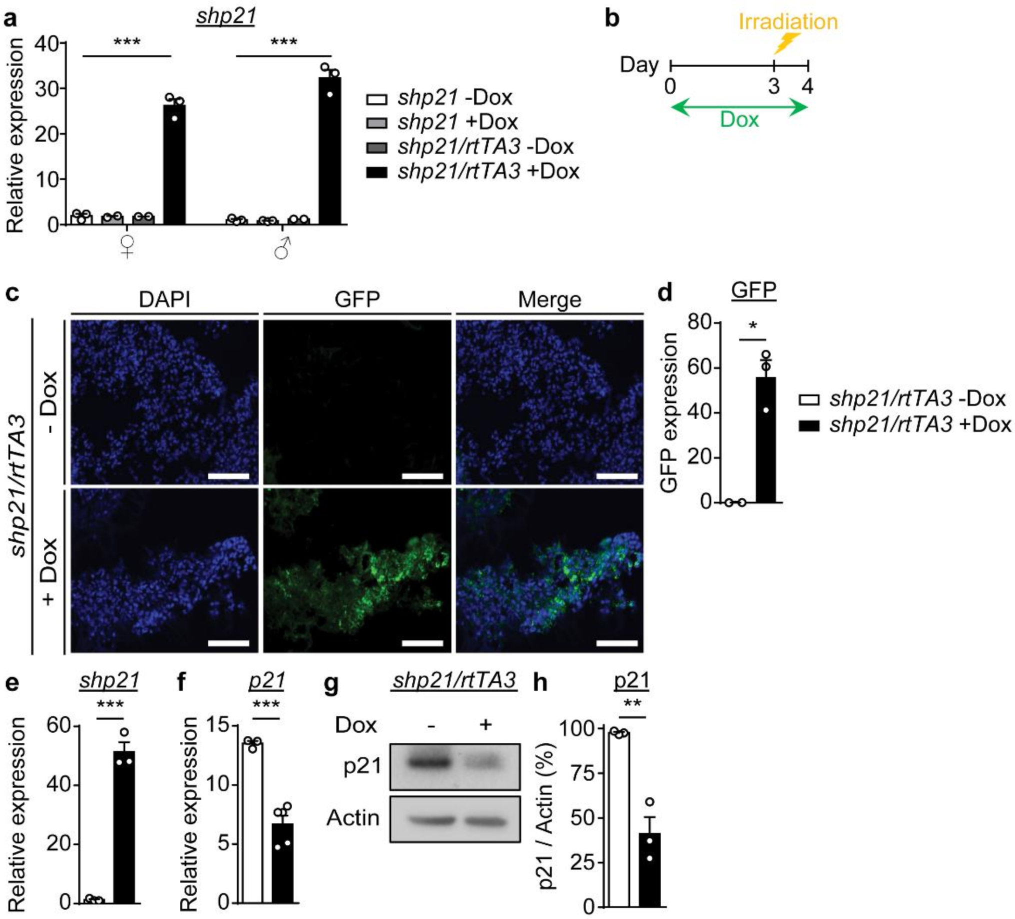

Appendix Figure S9: p21 knockdown is induced following Dox treatment in the lungs of CAG-rtTA3/tet-shp21 mice. (a) mRNA expression levels of *shp21* in lungs of double-transgenic *shp21/rtTA3* and transgenic *shp21* female (p=0.0001) and male (p=0.0001) mice treated with Dox for 3 days. (b) Experimental design of induction of p21 knockdown in *shp21/rtTA3* female mice. Mice were treated with Dox for 3 days and then irradiated (8Gy). Lungs were analyzed 1 day thereafter. (c) GFP florescence of lung sections from irradiated *shp21/rtTA3* mice described in (b). Scale bar, 200 µm. (d) Quantification of GFP levels presented in (c) (p=0.01). (e-f) mRNA expression levels of *shp21* (e) and *p21* (f) relative to control in the mice lungs described in (b) (*shp21*, p=0.0001; *p21*, p=0.0004). (g) Western blot analysis of p21 in the mice lungs described in (b). (h) Quantification of protein levels of p21 relative to GAPDH control presented in (g) (p=0.004). Data information: Data were analyzed using Student’s t-test. *P<0.05. **P<0.005. ***P<0.0005. Data are presented as mean ±SEM (a, n=2-3; b, n=5; c-d, n=3; e, n=3; f, n=3-5; g-h, n=3 independent repeats).

Appendix Figure S10

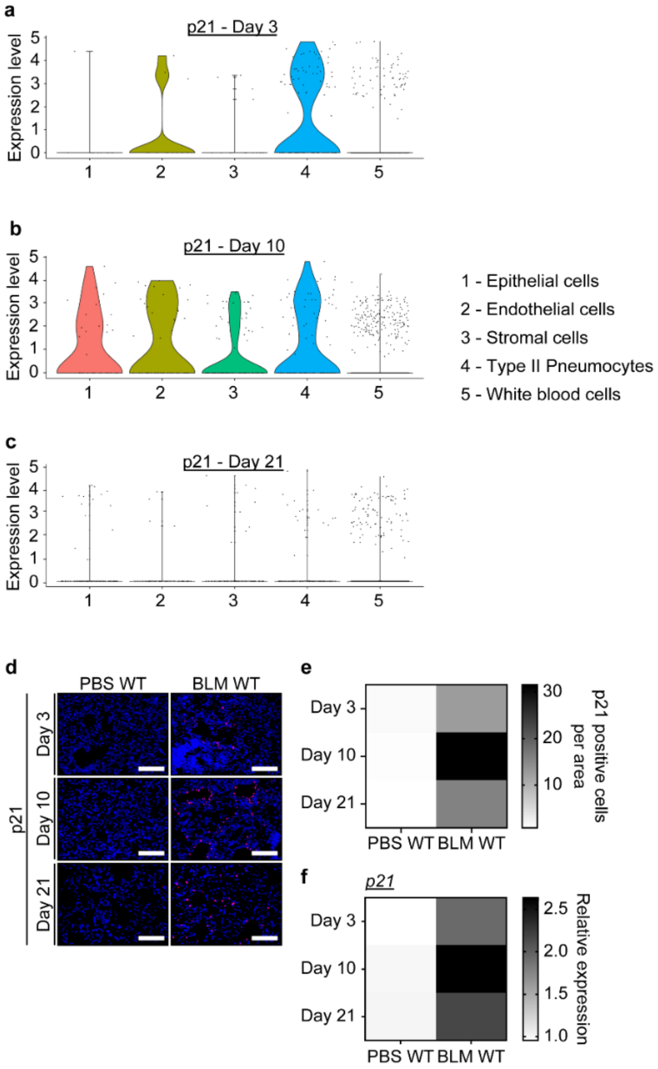

Appendix Figure S10: p21 expression at day 3, 10, and 21, in lung cells following BLM treatment. (a-c) Analysis of scRNA-seq data from Strunz et al. [1] (GEO accession code GSE141259) of female mice (WT) lung cells 3 days (n=3), 10 days (n=3), 14 and 21 days (n=4) following BLM or PBS vehicle administration (n=7). Data was reanalyzed to extract p21 expression in (1) Epithelial cells, (2) Endothelial cells, (3) Stromal cells, (4) Type II Pneumocytes and (5) White blood cells following bleomycin (BLM) administration. p21 expression was analyzed in these cell populations at (a) day 3, (b) day 10, and (c) day 21 post BLM treatment. (d) IF staining of p21 in WT mice that were administered with bleomycin (BLM) or PBS vehicle by one intra-tracheal installation. The lungs were analyzed 3 (upper panel), 10 (middle panel) and 21 (bottom panel) days post BLM administration. Scale bar: 200µm. (e) Quantification of p21 IF staining presented in (d). (f) mRNA expression levels of *p21* relative to control in the mice lungs described in (d). Data information: Data were analyzed using one-way ANOVA. *P<0.05. **P<0.005. ***P<0.0005. Data are presented as mean ±SEM (d-e, n=3-5; f, n=3-5 independent repeats).

Appendix Figure S11

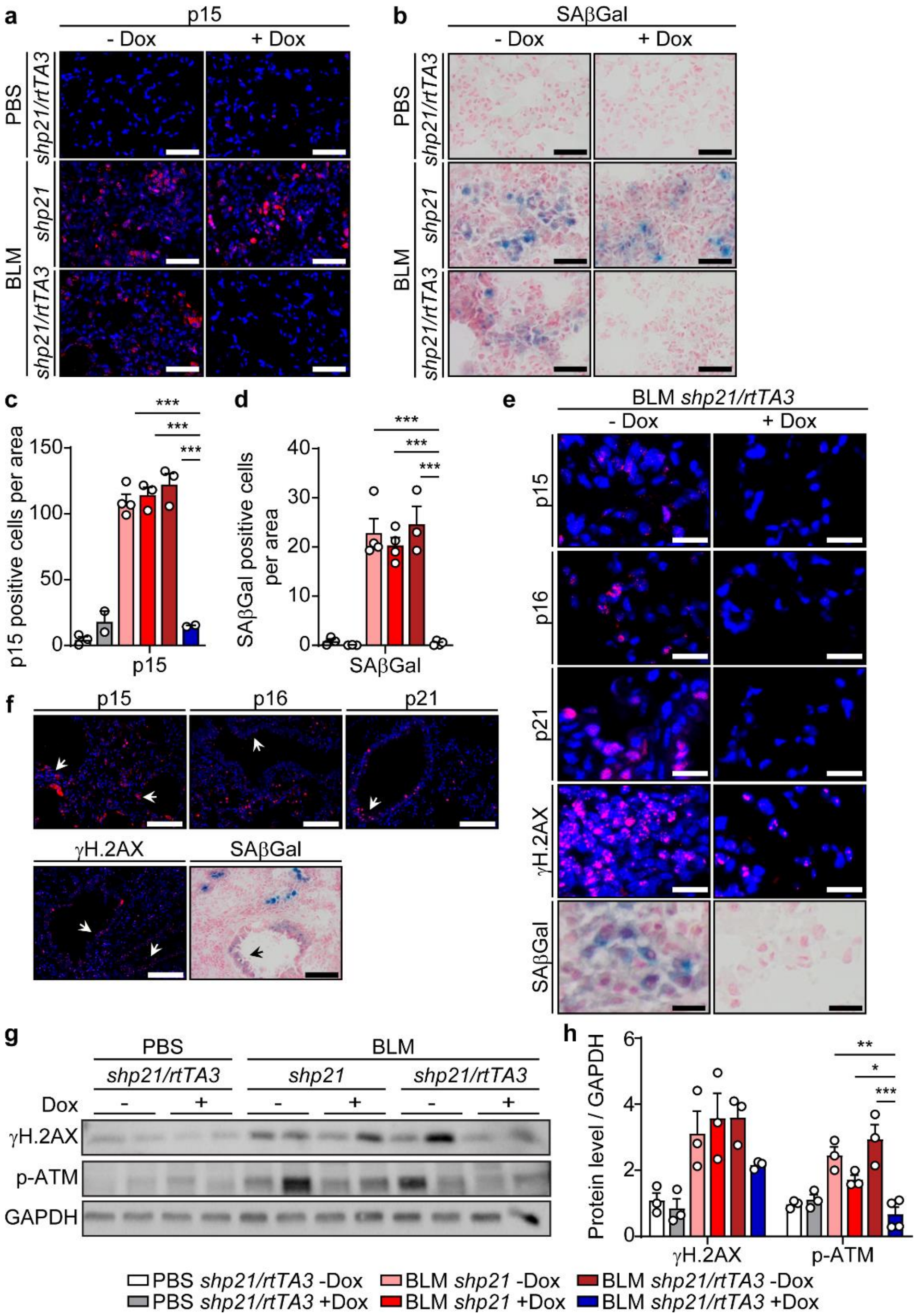

Appendix Figure S11: p21 knockdown leads to a reduction in the amount of senescent cells and protects from DDR in bleomycin-induced lung fibrosis. Related to Figure 5. (a) Lung sections from *shp21* and *shp21/rtTA3* mice that were administered once with bleomycin (BLM) or PBS vehicle treated with Dox 10 days thereafter for additional 11 days to activate *shp21*. Lungs were analyzed 21 days post BLM injection. Shown is an IF staining of p15. Scale bar: 100μm. (b)

SA-β-Gal staining of lung sections of the mice described in (a). Scale bar: 100µm. (c) Quantification of p15 IF staining presented in (a) (BLM *shp21/rtTA3* –Dox vs. BLM *shp21/rtTA3* +Dox, $p<0.0001$). (d) Quantification of SA-β-Gal staining presented in (b) (BLM *shp21/rtTA3* –Dox vs. BLM *shp21/rtTA3* +Dox, $p<0.0001$). (e) IF staining of p15, p16, p21, γH.2AX and a SA-β-Gal staining in *shp21/rtTA3* mice following BLM injection and treatment with Dox. Scale bar: 40µm. (f) IF staining demonstrates the bronchial epithelial cells staining of p15, p16, p21, γH.2AX and SA-β-Gal in *shp21/rtTA3* mice lungs following BLM injection. Scale bar: 200µm. (g) Western blot analysis of γH.2AX and phospho-ATM (p-ATM) in the mice lungs described in (a). (h) Quantification of protein levels of γH.2AX and p-ATM presented in (g) (BLM *shp21/rtTA3* –Dox vs. BLM *shp21/rtTA3* +Dox, pATM [$p=0.0001$]). Data information: Data were analyzed using one-way ANOVA. *$P<0.05$. **$P<0.005$. ***$P<0.0005$. Data represent mean ±SEM (a, c, n=2-4; b, d, n=3-4; e, n=3-4; f, n=3-4; g-h, n=3-4 independent repeats).

References

1. Strunz, M. *et al.* Alveolar regeneration through a Krt8+ transitional stem cell state that persists in human lung fibrosis. *Nature communications* **11**, 3559 (2020).
2. Saul, D. *et al.* A new gene set identifies senescent cells and predicts senescence-associated pathways across tissues. *Nature communications* **13**, 4827 (2022).
